# Doppler Waveform Alterations of the Supratesticular Artery and Associated Semen Biomarkers in Infertile Male Dromedary Camels

**DOI:** 10.3390/ani16020319

**Published:** 2026-01-20

**Authors:** Derar Derar, Ahmed Ali, Fahad A. Alshanbari, Mohammed H. Elzagafi

**Affiliations:** 1 Department of Clinical Sciences, College of Veterinary Medicine, Qassim University, Buraydah 51452, Saudi Arabia; dr.mohammad@qu.edu.sa (D.D.); ahme.ali@qu.edu.sa (A.A.); 2 Department of Medical Biosciences, College of Veterinary Medicine, Qassim University, Buraydah 51452, Saudi Arabia; 3 Department of Theriogenology, University Veterinary Hospital, Qassim University, Buraydah 51452, Saudi Arabia; melzagafi@qu.edu.sa

**Keywords:** male dromedary, fertility, biomarkers, testicular hemodynamics, diagnostic imaging

## Abstract

Male infertility in dromedary camels lacks objective diagnostic tools. This study evaluated the combined diagnostic value of testicular Doppler ultrasonography and semen biomarkers in 68 infertile (azoospermic, *n* = 21; oligozoospemic, *n* = 47) and 9 fertile male camels. All animals underwent a breeding soundness evaluation; computer-assisted semen analysis; color Doppler of the supratesticular artery; and a seminal plasma assessment for semenogelin I (SEM I), semenogelin II (SEM II), extracellular matrix protein 1 (ECM1), and testis-expressed protein 101 (TEX101). Infertile camels showed significantly impaired semen quality (*p* < 0.001). All four biomarkers were significantly lower in the infertile groups than controls (*p* = 0.001). Doppler indices indicated impaired testicular perfusion, with higher resistive and pulsatility indices (*p* = 0.003; *p* = 0.009) and lower velocity parameters (*p* < 0.001) in infertile animals. Biomarkers were strongly intercorrelated and negatively correlated with Doppler indices. ECM1 was the only significant predictor of infertility from the regression analysis (*p* = 0.031). Among the oligozoospemic camels stratified by motility, the >50% motility group had significantly higher SEM I and SEM II concentrations (*p* < 0.002). Integrating Doppler ultrasonography with biomarker profiling provides complementary diagnostic indicators for male camel infertility.

## Simple Summary

Male infertility in dromedary camels is a significant problem for breeders in arid regions, but current diagnostic methods are limited. This study investigated whether combining two advanced techniques—ultrasound measurement of testicular blood flow and analysis of specific protein biomarkers in semen—could improve the diagnosis of infertility in male camels. We examined 68 infertile camels and 9 fertile controls, measuring blood flow in the testicular arteries and levels of four fertility-related proteins in semen. Infertile camels showed reduced blood flow to the testes and lower levels of all four proteins compared with fertile animals. The protein ECM1 was particularly useful in identifying infertile animals. Camels with better sperm motility had higher levels of certain proteins. These findings suggest that combining ultrasound with biomarker testing provides a more comprehensive approach to diagnosing male infertility in camels, potentially helping breeders identify and manage reproductive problems more effectively.

## 1. Introduction

Infertility in male dromedary camels represents a significant yet often underappreciated constraint on herd productivity and reproductive efficiency. In the dromedary breeding system, failures in male fertility can manifest as sub-optimal sperm production, impaired copulation or libido, epididymal dysfunction, or testicular degeneration, all of which reduce conception rates, delay herd turnover, and increase maintenance costs per pregnancy [1,2,3]. Indeed, poor semen quality, arrested spermatogenesis, and testicular degeneration have been documented in infertile males of this species [4]. The economic impact is especially acute in arid and semi-arid production systems, where a single sire may serve many females such that a male with compromised fertility has disproportionate effects on the overall output [1,5,6]. Contributing factors may include infectious agents (e.g., Brucellosis, Trypanosomiasis), inbreeding, seasonality, age-related decline, and management stressors [3,7]. A notable proportion of infertility in male dromedary camels is linked to genetic predisposition, especially in presentations of primary infertility (impotentia generandi). Histopathological correlates in such cases frequently involve Sertoli cell-only syndrome [4]. This etiological pattern finds a parallel in human male infertility, where idiopathic cases constitute a significant and diagnostically challenging category [8,9]. Given the central role of male fertility in herd reproduction and the unique physiology of camels, improving diagnostic strategies for male infertility is essential for enhancing productivity.

Despite its importance, the body of literature on diagnostic approaches to male dromedary infertility remains relatively sparse. Traditional evaluation has largely relied on history, physical examination, semen analysis, and testicular biopsy [10]. For instance, a study evaluating breeding soundness in male dromedaries found that testicular ultrasonography and biopsy achieved sensitivities of 92.5% and 90%, respectively, when compared with semen analysis in infertile vs. fertile camels [1,11]. Three clinical forms of male infertility in camels, namely, post-coital infertility (IG), inability to copulate (IC), and lack of sexual desire (LSD), were investigated, and IG was linked to degenerative or spermatogenic disorders [2]. However, while seminal plasma biochemistry (e.g., trace elements) and molecular detection of pathogens have been studied (e.g., seminal plasma Zn, Cu, and Fe differences in infertile vs. fertile dromedaries [12]; molecular detection of Mycoplasma; and Leptospira in semen of infertile camels [3]), there remains a lack of advanced biomarker-driven and hemodynamic diagnostic approaches in this species. The field is thus poised for more sophisticated tools that allow for not just detection of infertility, but discrimination of underlying pathophysiology in males.

In humans and other animal species, seminal plasma and sperm-surface biomarkers have increasingly emerged as meaningful adjuncts to conventional semen analysis, offering insight into spermatogenic status, epididymal transit, obstruction, and functional competence [8,13]. Proteomic analyses have identified proteins such as semenogelin I (SEM I), semenogelin II (SEM II), extracellular matrix protein 1 (ECM1), and testis-expressed protein 101 (TEX101) as promising markers of male infertility [4,14]. Specifically, TEX101 (a testis-expressed germ-cell protein) and ECM1 (an extracellular matrix protein expressed in epididymis) have been validated in men for discriminating obstructive from non-obstructive azoospermia [15,16,17]. Likewise, SEMs I and II, major constituents of seminal plasma, have been reported as dysregulated in infertile men and varied semen parameter groups [18]. Their relevance speaks to molecular pathways underlying spermatogenesis, sperm maturation, and ejaculate physiology, and suggests potential cross-species translational value [19]. Extending these biomarker algorithms into production animals and camelids holds considerable promise for advancing male fertility diagnostics beyond traditional semen counts and motility.

Parallel to molecular biomarkers, ultrasound Doppler assessment of testicular and epididymal blood flow has gained traction as a non-invasive functional index of testicular health and spermatogenic output in men [15,16,20] and other animals [21,22,23]. In men with varicocele, increased testicular artery resistive index (RI), pulsatility index (PI), time-averaged maximum velocity (TAmax), time-averaged mean velocity (TAmean), and velocity time integral (VTI) have shown significant association with oligo-asthenospermia [24]. In bulls, the RI of the testicular artery correlated with sperm number, immature sperm, and morphological defects [22]. Although few studies exist in camels, one recent study on senile male camels treated with hCG demonstrated that decreased RI and PI (improved blood flow) correlated with improved semen quality and steroid levels [25]. This suggests that Doppler indices (such as RI, PI, TAmax, TAmean, and VTI) might serve as functional metrics of testicular perfusion, spermatogenic status, and overall male fertility. Incorporating such hemodynamic measures into camel reproductive clinics may improve diagnostic resolution and monitoring of treatment response [26].

Importantly, testicular vascular function and semen biomarker expression are biologically interconnected rather than independent diagnostic entities. Adequate testicular perfusion is essential for oxygen delivery, hormonal exchange, Sertoli cell activity, and germ cell metabolism, all of which directly influence spermatogenesis and post-testicular sperm maturation [19]. Compromised blood flow may therefore result in reduced synthesis, secretion, or release of fertility-associated proteins into seminal plasma [15]. Evaluating the correlation between Doppler-derived hemodynamic indices and semen biomarkers provides a mechanistic framework for understanding male infertility in dromedary camels as an integrated vascular–molecular dysfunction, rather than as isolated abnormalities in semen quality or protein expression [16].

Despite these promising avenues, research on the combined application of semen-biomarker profiling and Doppler vascular indices in male dromedary camels remains scarce. The camelid literature has largely focused on basic semen quality, seasonal effects, and collection methods, but the integration of molecular diagnostics and advanced imaging/hemodynamics in male fertility remains nascent. While proteomic biomarker work is extensive in humans [8,19], it has not yet been systematically applied in the dromedary context [4,27]. Similarly, Doppler studies in camels are limited to a few pilot investigations [25,26]. The paucity of data in this species therefore restricts our ability to stratify infertile males mechanistically, tailor therapeutic interventions, and develop evidence-based breeding soundness protocols specifically for camels.

In acknowledgement of this gap, the current research aims to evaluate the diagnostic significance of SEM I, SEM II, ECM1, and TEX101 biomarkers alongside Doppler-derived hemodynamic indices (RI, PI, TAmax, TAmean, and VTI) in male dromedary camels. By correlating these molecular and hemodynamic metrics with semen quality, spermatogenic status, and fertility outcomes, the study aspires to enhance early detection and differential diagnosis of male infertility in this species, provide normative data for Doppler parameters in camels, facilitate development of integrated diagnostic algorithms combining biomarker and imaging outputs, and offer robust tools to optimize male fertility management.

## 2. Materials and Methods

### 2.1. Animals, History, and Breeding Soundness Examination

A total of 68 male dromedary camels aged 4–15 years (6.13 ± 0.83 years) with documented histories of primary (failure of the male to impregnate females despite regular exposure to breeding or service, with no prior history of successful conception) or secondary (the inability to impregnate following one or more previous successful conceptions, as evidenced by a documented history of pregnancy in females or siring one or more offspring) infertility were presented to the University Veterinary Hospital, Qassim University, Saudi Arabia, for a comprehensive breeding soundness evaluation. The body condition scores (BCSs) varied between 3.5 and 4.5 on a 1–5 scale [28]. The mean duration of infertility was 31.6 ± 25.9 months (11–45 months). In addition, a reference group (control; *n* = 9) of fertile males (selected based on documented evidence of successful conception in females during the preceding breeding season, in addition to normal libido, mating behavior, and semen characteristics within established reference ranges) aged 6–12 years (7.62 ± 3.51 years) with BCSs ranging from 3 to 4 was included for comparison.

The breeding soundness assessment commenced with a detailed anamnesis, focusing on libido, mating ability, previous reproductive performance, medical history, traumatic injuries, and prior pharmacological treatments. Breeding records indicated that all enrolled animals exhibited normal sexual drive, appropriate copulatory performance, and a consistent willingness to mate when exposed to receptive females [29]. Observed sexual behavior was typical for the species and included necking, olfactory investigation, mounting following induction of female squatting, and maintenance of the normal mating posture. All males achieved and sustained a penile erection throughout copulation until ejaculation, followed by normal dismounting.

Each camel underwent a complete general clinical examination, in addition to detailed evaluation of the external and internal genital organs and ultrasonographic assessment using a Sonoscape X3V ultrasound unit (Sonoscape Medical Corp., Hamburg, Germany) equipped with a 5–10 MHz interchangeable linear-array transducer. All examinations were performed in accordance with previously established and validated protocols [4].

### 2.2. Semen Collection and Analysis

Semen samples were obtained from all animals using an electroejaculation technique with a commercial electroejaculator (ElectroJac 6©, Neogen, Lexington, KY, USA), following procedures previously described for dromedary camels [4]. Immediately after collection, ejaculates were placed in sterile, capped glass containers and maintained at ambient temperature (25–35 °C) for approximately 30 min to allow for complete liquefaction. During this period, samples were protected from direct light, excessive agitation, and prolonged air exposure, following established camel semen handling protocols [11,12].

All semen assessments were conducted by a single trained examiner to minimize inter-observer variability. Ejaculates were evaluated for physical and functional characteristics, including the sperm concentration, individual progressive motility, viability, and morphology, using a computer-assisted semen analysis (CASA) system (AndroVision^®^ CASA, Ref. No. 12500/0000; Minitube International GmbH, Tiefenbach, Germany). For the motility assessment, semen samples were diluted 1:1 with a laboratory-prepared sodium citrate buffer (2.9% *w*/*v*; 29 g sodium citrate per liter of distilled water), adjusted to pH 6.8–7.0. The osmolality of the buffer was approximately 290–310 mOsm/kg, corresponding to physiological conditions for camel spermatozoa. The buffer was freshly prepared and maintained at ambient temperature prior to use. A drop of the diluted sample was placed on a pre-warmed glass slide and examined under ×40 magnification using a phase contrast microscope, as previously reported [30]. Sperm viability (percentage of viable sperm) was assessed using eosin–nigrosin staining, with viable spermatozoa denying eosin uptake; at least 200 sperm cells were evaluated per sample. The sperm morphology was evaluated on air-dried semen smears stained with a methyl violet stain [4]. For each animal, the device generates a dedicated file containing the microscopic analysis (count, motility, morphology) of the semen sample.

Classification of the semen quality parameters was performed according to previously established criteria [2]. Based on semen analysis outcomes, animals were categorized as azoospermic (*n* = 21) or oligozoospemic (*n* = 47). Doppler ultrasonographic variables and semen biomarker data obtained from these groups were compared with those recorded in fertile male dromedaries (control group; *n* = 9).

To further investigate the relationship between sperm motility impairment, testicular blood perfusion (TBP) parameters, and semen biomarkers, oligozoospemic males were subdivided according to the percentage of progressive motility into four categories: 0% (*n* = 11), 1–30% (*n* = 13), 31–50% (*n* = 12), and >50% (*n* = 11). These subgroups were subsequently used to assess the diagnostic performance of TBP indices and semen biomarkers in detecting varying degrees of motility deterioration.

### 2.3. Color Doppler Examination

Color and spectral Doppler ultrasonography of the supratesticular artery was performed following previously described and validated methodologies in camelids and other domestic species, where Doppler indices have been shown to reflect testicular perfusion and spermatogenic function [21,22,25,26,31]. Evaluation of testicular blood perfusion (TBP) was performed using color Doppler ultrasonography on camels who were adequately restrained in a sternal recumbent (sitting) position during examination, with the hind limbs folded beneath the body. An elevating crane was used to support and stabilize the animal when necessary, allowing for an optimal and safe approach to the scrotum for proper access of the supratesticular arteries. Assessment of testicular hemodynamics was conducted at the level of the supratesticular artery (STA), following previously described techniques [25,26]. The TBP assessment was achieved by activating the spectral Doppler mode until a clear and stable spectral waveform of blood flow within the target vessel was obtained. Doppler velocity parameters were then measured and recorded for subsequent analysis. To ensure methodological consistency, all examinations were conducted by the same operators, at standardized times of the day (09:00–10:00 h), using identical machine settings throughout the study.

For each measurement, at least three consecutive cardiac cycles exhibiting comparable systolic peak amplitude and velocity were selected for analysis. The pulsatility index (PI), resistive index (RI), time-averaged maximum velocity (Tmax), time-averaged mean velocity (Tmean), and velocity time integral (VTI) were automatically calculated using the built-in Doppler software (https://www.sonoscape.com/en/products/veterinary/ultrasound_solutions/portable_color_doppler/2022/1107/145.html?utm_source=chatgpt.com, accessed on 22 December 2025). To minimize the technical variability, ultrasonographic parameters were maintained constant for all examinations, including B-mode frequency (10 MHz), color Doppler frequency (5 MHz), color gain (28%), color pulse repetition frequency (1 kHz), pulsed-wave Doppler frequency (5 MHz), pulsed-wave gain (70%), and pulsed-wave pulse repetition frequency (2 kHz).

### 2.4. Seminal Plasma Collection and Semen Biomarkers Analysis

Following the semen collection and gross and microscopic evaluations, the semen of the affected and control animals was centrifuged at 1700× *g*. Seminal plasma was separated and stored at −20 °C until the time it was assayed for concentrations the semen biomarkers. Seminal plasma samples were subsequently analyzed for the semen-related biomarkers semenogelin I (SEM I, catalog number MBS047619), semenogelin II (SEM II, catalog number, MBS089734), testis-expressed protein 101 (TEX101, catalog number MBS109178), and extracellular matrix protein 1 (ECM1, catalog number 074772). Quantitative determination of the biomarker concentrations was performed using enzyme-linked immunosorbent assay (ELISA) kits specifically designed for these analytes (MyBioSource Inc., San Diego, CA, USA). All assays were performed in duplicate, and absorbance was measured using a microplate reader (Multiskan GO, Thermo Fisher Scientific, Waltham, MA, USA) at the recommended wavelength in accordance with the manufacturer’s instructions. The intra-assay coefficients of variation ranged from 4.9 to 8.3% for SEM I, 5.9 to 7.5% for SEM II, 3.7 to 7.4% for TEX101, and 4.5 to 8.2% for ECM1, indicating acceptable assay precision. The analytical sensitivity of each assay was 10 pg/mL for the evaluated biomarkers.

### 2.5. Statistical Analysis

Statistical analyses were conducted using SPSS version 15, SPSS Inc., Chicago, IL, USA. Data normality was assessed using the Shapiro–Wilk test, and homogeneity of variances was evaluated with Levene’s test. For normally distributed variables with equal variances, one-way analysis of variance (ANOVA) was performed to compare means across the fertile, azoospermic, and oligozoospemic groups. When the ANOVA indicated significant differences, post hoc comparisons were carried out using Tukey’s honestly significant difference (HSD) test. For variables violating the assumption of equal variances, the Games–Howell post hoc test was applied. Non-normally distributed data were analyzed using the Kruskal–Wallis test, followed by Dunn’s post hoc procedure. Relationships between continuous variables were examined with Pearson’s correlation coefficient for normally distributed data and Spearman’s rank correlation for non-normally distributed data. To identify predictors of group membership, multiple linear regression was employed with the four semen biomarkers as independent variables. A *p*-value of <0.05 was considered statistically significant.

## 3. Results

Compared with the control group, the oligozoospemic camels exhibited a significant reduction in sperm motility (21.65 ± 0. 31 vs. 57.5 ± 15.8%) (*p* < 0.001). Sperm abnormality (55.0 ± 27.3 vs. 26.9 ± 6.5%) and percentage of dead sperms (73.5 ± 26.8 vs. 24.4 ± 12.1%) were significantly higher in the oligozoospemic camels than in the controls (*p* < 0.001). The levels of SEM I, SEM II, ECM1, and TEX101 differed significantly between the control, azoospermic, and oligozoospemic camels (*p* = 0.001). Both infertile groups exhibited significantly lower biomarker levels compared with the control group (Table 1 and Supplementary Materials).

**Figure 1:**
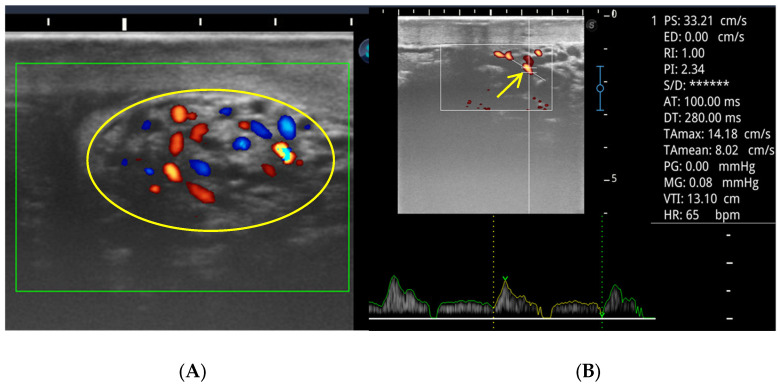
Color flow mapping (CFM) of the pampiniform plexus (yellow ellipse; blue colored-blood vessels are those moving away and red colored-blood vessels are those moving toward the transducer) in a male camel (**A**) and pulsed-wave Doppler waveform of the supratesticular artery (arrow), demonstrating the spectral flow pattern used for hemodynamic parameter calculation (PS, peak systole; ED, end diastole; RI, resistive index; PI, pulsatility index, S/D, PS/ED; AT, Acceleration Time, DT, Deceleration Time; TAmax, Time-Averaged Maximum Velocity; TAmean, Time-Averaged Mean Velocity; PG, Peak Pressure Gradient; MG, Mean Pressure Gradient; VTI, Velocity–Time Integral; HR, heart rate (**B**).

Figure 1 illustrates representative color flow mapping of the pampiniform plexus and the corresponding pulsed-wave Doppler waveform of the supratesticular artery obtained during imaging analysis and used for calculation of hemodynamic parameters.

Mean values of the RI and PI (Table 2) showed significant differences between the three groups (*p* = 0.003 and *p* = 0.009, respectively). In addition, TAmax, TAmean, and VTI differed significantly between the groups (*p* < 0.001).

Using linear regression to distinguish infertility, among the four biomarkers examined, only ECM was a statistically significant predictor (B = −0.209, SE = 0.094, β = −0.363, *p* = 0.031), indicating a negative association with azoospermic and oligozoospermic groups (Supplementary Materials). The predictors SEM I (*p* = 0.246), SEM II (*p* = 0.355), and TEX101 (*p* = 0.958) were not statistically significant. As in Table 3, strong positive intercorrelations were observed between the semen biomarkers SEM I, SEM II, ECM, and TEX101 (r = 0.75–0.84, *p* < 0.01). All semen biomarkers were negatively correlated with both Doppler indices (RI: r = −0.39 to −0.45, PI: r = −0.42 to −0.48, *p* < 0.01). The resistive and pulsatility indices were strongly positively correlated (r = 0.91, *p* < 0.01). Fertility group correlations revealed that higher SEM I and SEM II levels were significantly associated with the control group (r = 0.64 and 0.68, respectively, *p* < 0.01), whereas lower levels correlated with the oligo group (r = −0.35 and −0.38, *p* < 0.05). ECM and TEX101 were not significantly correlated with any group. The RI was negatively correlated with the oligo group (r = −0.33, *p* < 0.05). All other group correlations were non-significant (*p* > 0.05).

**Figure 2:**
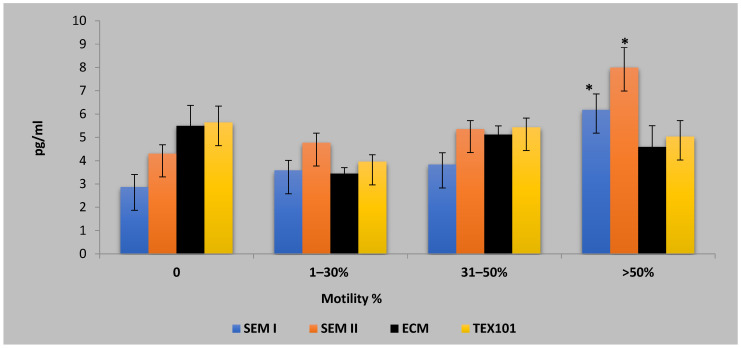
Concentrations of seminal plasma biomarkers—semenogelin I (SEM I), semenogelin II (SEM II), extracellular matrix protein 1 (ECM1), and testis-expressed protein 101 (TEX101) (pg/mL)—in asthenozoospermic dromedary camels classified according to sperm motility percentage. Data are presented as mean ± SEM. * Indicates values that are significantly different between motility categories (*p* < 0.05).

The testicular Doppler parameters were compared across four sperm motility groups (0%, 1–30%, 31–50%, and >50%). No statistically significant differences were observed between the groups for any of the Doppler measures. Figure 2 presents the concentrations of semen biomarkers across the motility-based asthenozoospermic categories. Significant differences were observed for SEM I and SEM II in the high-motility group (>50%), with *p*-values of 0.002 and 0.001, respectively. ECM and TEX101 showed less pronounced variation across the motility categories. Post hoc analyses identified specific group differences in sperm parameters and semen biomarkers across the motility categories. Sperm abnormality was significantly lower in the >50% motility group compared with both the 1–30% group (*p* = 0.006) and the 31–50% group (*p* = 0.007). The percentage of dead sperms was significantly higher in the 0% motility group compared with the 31–50% (*p* = 0.029) and >50% groups (*p* = 0.002). The SEM I and SEM II levels were significantly elevated in the high-motility group (>50%) compared with all lower-motility categories (all *p* < 0.05). No other pairwise comparisons were statistically significant. 

Significant correlations between the study parameters are summarized in Table 4. Very strong positive correlations were observed between the semen biomarkers (SEM I–SEM II: r = 0.957, *p* < 0.001; ECM–TEX101: r = 0.952, *p* < 0.001) and between the Doppler indices (RI–PI: r = 0.797, *p* < 0.001; TAmax–TAmean: r = 0.940, *p* < 0.001). Moderate negative correlations were identified between the sperm quality markers and semen biomarkers, including abnormality–SEM I (r = −0.530, *p* = 0.005) and percentage of dead sperms–SEM II (r = −0.579, *p* = 0.002). Cross-domain correlations were also noted, such as moderate negative relationships between the PI and TAmax (r = −0.555, *p* = 0.003) and between TEX101 and VTI (r = −0.568, *p* = 0.002).

## 4. Discussion

The present study provides an integrated evaluation of testicular hemodynamics and semen-related biomarkers in infertile male dromedary camels, offering novel insights into the vascular–molecular interplay underlying impaired spermatogenesis in this species. By combining the color Doppler ultrasonography of the supratesticular artery with the serum biomarker profiling, the findings extend conventional breeding soundness examination and contribute objective diagnostic indicators for camel male infertility.

Infertile camels, particularly those classified as oligozoospemic, exhibited marked deterioration in semen quality, characterized by significantly reduced sperm concentration and motility and increased sperm abnormalities and mortality. These findings are consistent with previous reports describing impaired spermatogenesis, testicular degeneration, and defective sperm maturation as central features of male infertility in dromedary camels [2,4]. The magnitude of sperm quality deterioration observed in the present study further underscores the biological and economic consequences of male infertility in camel production systems, where a single sire often services multiple females [1,5,6].

A key finding of this study is the significant alteration in testicular blood perfusion (TBP) among infertile camels, as evidenced by the elevated RI and PI and concomitant reductions in velocity-based Doppler parameters. Increased RI and PI reflect elevated downstream vascular resistance and reduced tissue perfusion, which may compromise oxygen and nutrient delivery to the testicular parenchyma [31]. Similar associations between increased Doppler indices and impaired semen quality have been reported in infertile men with oligoasthenospermia [20,24] and in domestic animals, including bulls [22] and stallions [21]. In camelids, available data remain limited; however, recent work demonstrated that improvement in testicular perfusion following hormonal stimulation was associated with enhanced semen quality and endocrine status in senile camels [25]. Collectively, these findings support the hypothesis that testicular vascular dysfunction contributes to impaired spermatogenesis in camels and highlight Doppler ultrasonography as a valuable non-invasive indicator of testicular functional status [26,31].

All evaluated semen-related biomarkers—SEM I, SEM II, TEX101, and ECM1—were significantly reduced in the azoospermic and oligozoospemic camels compared with the fertile controls. This pattern aligns with earlier camel studies reporting altered seminal plasma proteins in association with infertility and asthenozoospermia [4]. Semenogelins I and II are major constituents of seminal plasma involved in sperm motility regulation and ejaculate physiology, and their reduced levels in infertile camels likely reflect disrupted accessory gland and sperm–plasma interactions, as previously described in humans and livestock species [13,18,19]. The observed reduction in TEX101 and ECM1 is particularly noteworthy. In human medicine, these biomarkers have been validated as discriminators between obstructive and non-obstructive azoospermia and as indicators of spermatogenic activity and epididymal function [17]. Their downregulation in infertile camels suggests a conserved biological role across species and supports their translational relevance in camel reproductive diagnostics, as recently emphasized in proteomic overviews of livestock reproduction [27].

Stratification of oligozoospemic camels according to sperm motility percentage revealed a clear, graded association between semen biomarkers, testicular hemodynamics, and sperm functional competence. Progressive improvement in motility was accompanied by stepwise increases in SEM I, SEM II, ECM1, and TEX101 concentrations, while lower-motility categories were characterized by elevated Doppler resistance indices (RI and PI) and reduced velocity parameters. These findings indicate that deterioration in sperm motility within the oligozoospemic population reflects an underlying continuum of vascular and molecular dysfunction rather than a binary fertile–infertile state [8,14]. The strong negative correlations between Doppler resistance indices and semen biomarkers support a close functional link between impaired testicular perfusion and reduced expression or release of fertility-associated proteins, suggesting compromised Sertoli cell function, germ cell metabolism, and post-testicular maturation under conditions of reduced blood flow [15,16,24].

Among the evaluated biomarkers, ECM1 demonstrated the strongest association with the motility-based categorization and emerged as a significant predictor of infertility, emphasizing its diagnostic relevance in oligozoospemic camels. Given that ECM1 is predominantly expressed in the epididymis, its progressive decline across lower-motility categories suggests that epididymal dysfunction contributes substantially to reduced sperm motility and viability, complementing the effects of altered testicular hemodynamics [19]. The motility-dependent biomarker patterns together with the Doppler flow characteristics support a unified pathophysiological model in which vascular compromise, impaired epididymal maturation, and biochemical alterations act synergistically to determine the severity of reproductive dysfunction in oligozoospemic dromedary camels, as indicated in men [15,16,32] and other livestock [21,22]. Although the Doppler parameters did not differ significantly between the motility-based oligozoospemic subgroups, SEM I and SEM II were significantly elevated in the high-motility males (>50%). This finding suggests that semenogelins are more sensitive to functional sperm performance than Doppler indices alone, reinforcing their utility as adjunct markers for assessing sperm competence [18,20,25]. The absence of Doppler differences between motility subgroups may reflect compensatory vascular mechanisms or the multifactorial nature of motility impairment [31,33].

Clinically, the combined use of Doppler ultrasonography and seminal plasma biomarker profiling could enhance infertility diagnosis in camels, allowing for more targeted interventions, such as hormonal therapy, nutritional management, or surgical correction of vascular abnormalities. This integrated approach may improve reproductive management in breeding programs and help identify subfertile males earlier in their reproductive lifespan. While the findings demonstrate the diagnostic potential of seminal plasma biomarkers, their routine application in every case of subfertility must be considered within a practical clinical framework. The decision to employ biomarker profiling should be guided by a cost–benefit analysis, weighing factors such as the individual animal’s breeding value, owner’s financial constraints, the availability of specialized reagents, and the specific clinical presentation. Biomarker assessment is most warranted in cases of idiopathic infertility or when conventional diagnostics are inconclusive, thereby offering targeted insights where traditional methods fall short.

The study’s findings should be interpreted in light of certain confines. The cross-sectional design establishes associations but cannot confirm causality. The sample sizes, particularly for the fertile control group, were limited, which may affect statistical power and generalizability. Differences in the body condition, age, or subclinical health status between subjects could represent confounding variables not fully controlled for. Additionally, the single timepoint assessments do not account for potential seasonal or physiological variations in testicular hemodynamics or biomarker expression. Future longitudinal studies with larger cohorts are warranted to validate these parameters as reliable diagnostic tools.

## 5. Conclusions

Taken together, the present findings support the combined application of color Doppler ultrasonography and semen biomarker profiling as complementary diagnostic tools for male infertility in dromedary camels. This integrated approach may enhance early detection, facilitate mechanistic stratification of infertility phenotypes, and guide targeted therapeutic or management interventions. Further longitudinal studies are warranted to establish biomarker cut-off values, evaluate treatment responsiveness, and validate these tools under field conditions.

## Figures and Tables

**Table 1:** Level of semenogelin I (SEM I), semenogelin I (SEM II), extracellular matrix protein 1 (ECM1), and testis-expressed protein 101 (TEX101) in azoospermic and oligozoospermic camels compared with control fertile camels.

Biomarker (pg/mL)	Control (*n* = 9)	Azoospermic (*n* = 21)	Oligozoospemic (*n* = 47)	*p*-Value
SEM I	6.10 ± 1.73 a	3.18 ± 0.62 b	3.42 ± 0.96 b	0.001
SEM II	7.41 ± 2.35 a	3.88 ± 0.84 b	4.08 ± 0.63 b	0.001
ECM	6.27 ± 1.33 a	4.19 ± 1.19 b	3.87 ± 0.77 b	0.001
TEX101	6.58 ± 1.72 a	3.62 ± 0.86 b	3.82 ± 0.66 b	0.001

Values with different letters in the same row are significantly different. *p*-value was set at 0.05.

**Table 2:** Testicular Doppler hemodynamic parameters, including resistive index (RI), pulsatility index (PI), time-averaged maximum velocity (TAmax), time-averaged mean velocity (TAmean), and velocity time integral (VTI), in fertile, azoospermic, and oligozoospemic dromedary camels.

Parameter	Control (*n* = 9)	Azoospermia (*n* = 21)	Oligozoospermia (*n* = 47)	*p*-Value
RI	0.25 ± 0.13 a	0.69 ± 0.30 b	0.52 ± 0.30 ab	0.003
PI	0.31 ± 0.21 a	1.62 ± 0.94 b	0.93 ± 1.10 ab	0.009
TAmax (cm/s)	13.39 ± 4.00 a	2.50 ± 0.53 b	11.82 ± 6.31 a	<0.001
TAmean (cm/s)	8.74 ± 1.90 a	1.20 ± 0.32 b	7.01 ± 4.14 a	<0.001
VTI (cm)	20.07 ± 5.27 a	2.50 ± 0.43 b	18.81 ± 14.29 a	<0.001

Values expressed in means ± SE. Values with different letters in the same row are significantly different. *p*-value was set at 0.05.

**Table 3:** Correlations between semen biomarkers [semenogelin I (SEM I), semenogelin I (SEM II), extracellular matrix protein 1 (ECM1), and testis-expressed protein 101]; Doppler ultrasound indices [resistive index (RI), pulsatility index (PI)]; and control fertile, azoospermic, and oligozoospermic male dromedaries.

Variable	SEM I	SEM II	ECM	TEX101	Resistive Index	Pulsatility Index	Control	Azoo	Oligo
SEM I	--	0.82 **	0.78 **	0.80 **	−0.42 **	−0.46 **	0.64 **	−0.25	−0.35 *
SEM II	0.82 **	--	0.75 **	0.84 **	−0.45 **	−0.48 **	0.68 **	−0.30 *	−0.38 *
ECM	0.78 **	0.75 **	--	0.79 **	−0.39 **	−0.42 **	−0.04	0.22	−0.18
TEX101	0.80 **	0.84 **	0.79 **	--	−0.41 **	−0.44 **	−0.00	0.18	−0.15
Resistive Index	−0.42 **	−0.45 **	−0.39 **	−0.41 **	--	0.91 **	0.23	0.10	−0.33 *
Pulsatility Index	−0.46 **	−0.48 **	−0.42 **	−0.44 **	0.91 **	--	0.12	0.05	−0.17

** *p* < 0.01, * *p* < 0.05.

**Table 4:** Correlations between semen biomarkers [semenogelin I (SEM I), semenogelin I (SEM II), extracellular matrix protein 1 (ECM1), testis-expressed protein 101 (TEX101)], hemodynamic parameters [resistive index (RI), pulsatility index (PI), time-averaged maximum velocity (TAmax), time-averaged mean velocity (TAmean), and velocity time integral (VTI)] for the different groups of asthenozoospermic camels classified according to the motility score.

Correlation	Pearson’s r (95% CI)	*p*-Value	Strength
Between Semen Biomarkers			
SEM I–SEM II	0.957 (0.905, 0.982)	<0.001	Very strong
ECM–TEX101	0.952 (0.894, 0.979)	<0.001	Very strong
Between Doppler Parameters			
Resistive–pulsatility indices	0.797 (0.603, 0.904)	<0.001	Strong
TAmax–TAmean	0.940 (0.868, 0.974)	<0.001	Very strong
TAmax–VTI	0.725 (0.469, 0.871)	<0.001	Strong
TAmean–VTI	0.719 (0.459, 0.867)	<0.001	Strong
Between Semen Quality and Biomarkers			
Abnormality–SEM I	−0.530 (−0.768, −0.163)	0.005	Moderate
Abnormality–SEM II	−0.586 (−0.798, −0.240)	0.002	Moderate
Percentage of dead sperms–SEM I	−0.559 (−0.785, −0.197)	0.003	Moderate
Percentage of dead sperms–SEM II	−0.579 (−0.796, −0.229)	0.002	Moderate
Abnormality–percentage of dead sperms	0.573 (0.257, 0.783)	0.002	Moderate
Cross-domain Correlations			
Pulsatility index–TAmax	−0.555 (−0.783, −0.190)	0.003	Moderate
Pulsatility index–TAmean	−0.517 (−0.761, −0.144)	0.007	Moderate
ECM–VTI	−0.480 (−0.739, −0.097)	0.013	Moderate
TEX101–VTI	−0.568 (−0.790, −0.209)	0.002	Moderate

## Data Availability

The raw data supporting the conclusions of this article will be made available by the authors, without undue reservation.

## Supplementary Materials

The following supporting information can be downloaded from https://www.mdpi.com/article/10.3390/ani16020319/s1: Table S1. Clinical and semen parameters; Table S2. Post hoc comparisons of semen biomarkers (mean differences); Table S3. Linear regression predicting fertility group from biomarker; Table S4. Test of homogeneity of variances (Levene’s test); Table S5. Normality test results (Shapiro–Wilk); Table S6. Post hoc analysis showing specific group differences; Table S7. Significant correlations between studied parameters for the different groups classified according to the motility score.
