# Supplementary material for: Doppler Waveform Alterations of the Supratesticular Artery and Associated Semen Biomarkers in Infertile Male Dromedary Camels
Source: Animals (Basel). 2026 Jan 20;16(2):319. doi: 10.3390/ani16020319 (PMC12837927; doi:10.3390/ani16020319)
Supplement: Supplementary file 1 [file animals-16-00319-s001.zip › animals-4088481-supplementary.pdf]

Table S1: Clinical and Semen Parameters

Parameter	Control (n=8)	Azoospermia (n=15)	Oligospermia (n=26)	p-value
**Sperm Concentration (×10³/mL)**	475 ± 65.5a	0 ± 0b	214.1 ± 83.2c	<0.001
**Motility (%)**	57.5 ± 15.8a	0 ± 0b	21.65 ± 0. 31b	<0.001
**Abnormality (%)**	26.9 ± 6.5a	0 ± 0b	55.0 ± 27.3c	<0.001
**Percentage of dead sperms (%)**	24.4 ± 12.1a	0 ± 0b	73.5 ± 26.8c	<0.001

Table S2: Post-Hoc Comparisons of Semen Biomarkers (Mean Differences)

Comparison	SEM I	SEM II	ECM	TEX101
Fertile vs. Azoo	2.92 ± 0.45*	3.53 ± 0.49*	2.07 ± 0.44*	2.96 ± 0.42*
Fertile vs. Oligo	2.68 ± 0.42*	3.33 ± 0.46*	2.39 ± 0.41*	2.76 ± 0.39*
Azoo vs. Oligo	0.24 ± 0.34	0.20 ± 0.37	0.32 ± 0.33	0.19 ± 0.31

Values are mean differences ± standard error.

*** p < 0.05 (Tukey's HSD).**

Table S3: Linear Regression Predicting Fertility Group from Biomarker

Predictor	Unstandardized B	SE	β	t	p-value
Constant	4.079	0.312	–	13.075	<0.001
SEM I	-0.112	0.095	-0.215	-1.175	0.246
SEM II	-0.089	0.095	-0.198	-0.935	0.355
**ECM**	**-0.209**	0.094	**-0.363**	**-2.226**	**0.031**
TEX101	0.007	0.127	0.013	0.053	0.958

Model summary: $R = 0.660$, $R^2 = 0.435$, Adjusted $R^2 = 0.384$, $F(4,44) = 8.471$, $p < 0.001$.

Bold indicates statistical significance at p < 0.05.

Table S4: Test of Homogeneity of Variances (Levene's Test)

Variable	Levene Statistic	df1	df2	p-value
SEM I	6.162	2	46	0.004
SEM II	14.565	2	46	<0.001
ECM	1.467	2	46	0.241
TEX101	8.797	2	46	0.001
Resistive Index	5.619	2	46	0.007
Pulsatility Index	3.301	2	46	0.046

Significant p-values (<0.05) indicate violation of homogeneity of variances.

Table S5: Normality Test Results (Shapiro-Wilk)

Variable / Group	Fertile (p)	Azoo (p)	Oligo (p)
SEM I	0.680	0.674	0.092
SEM II	0.508	0.369	0.874
ECM	0.276	0.446	0.205
TEX101	0.139	0.327	0.583
Resistive Index	0.045*	<0.001*	0.066
Pulsatility Index	0.010*	<0.001*	<0.001*

Table S6: Post-Hoc Analysis Showing Specific Group Differences

Significant Parameter	Group Comparison	Mean Difference ± SE	p-value	Interpretation
Abnormality	0% vs 31-50%	34.17 ± 11.93	0.042	a vs b
	1-30% vs >50%	44.17 ± 11.93	0.006	a vs b
	31-50% vs >50%	40.63 ± 11.16	0.007	a,b vs b
Percentage of dead sperms	0% vs 31-50%	36.67 ± 12.07	0.029	a vs b
	0% vs >50%	50.00 ± 12.07	0.002	a vs b
SEM I	>50% vs 0%	3.31 ± 0.78	0.002	b vs a
	>50% vs 1-30%	2.60 ± 0.73	0.009	b vs a
	>50% vs 31-50%	2.35 ± 0.78	0.031	b vs a
SEM II	>50% vs 0%	3.70 ± 0.78	0.001	b vs a
	50% vs 1-30%	3.22 ± 0.73	0.001	b vs a
	>50% vs 31-50%	2.64 ± 0.78	0.013	b vs a

Table S7: Significant correlations among studied parameters for the different groups classified according to the motility score

Correlation	Pearson's r (95% CI)	p-value	Strength
Between Semen Biomarkers			
SEM I - SEM II	0.957 (0.905, 0.982)	<0.001	Very strong
ECM - TEX101	0.952 (0.894, 0.979)	<0.001	Very strong
Between Doppler Parameters			
Resistive - Pulsatility Index	0.797 (0.603, 0.904)	<0.001	Strong
TA Max Vel - TA Mean Vel	0.940 (0.868, 0.974)	<0.001	Very strong
TA Max Vel - VTI	0.725 (0.469, 0.871)	<0.001	Strong
TA Mean Vel - VTI	0.719 (0.459, 0.867)	<0.001	Strong
Between Semen Quality & Biomarkers			
Abnormality - SEM I	-0.530 (-0.768, -0.163)	0.005	Moderate
Abnormality - SEM II	-0.586 (-0.798, -0.240)	0.002	Moderate
Percentage of dead sperms - SEM I	-0.559 (-0.785, -0.197)	0.003	Moderate
Percentage of dead sperms - SEM II	-0.579 (-0.796, -0.229)	0.002	Moderate
Abnormality - Percentage of dead sperms	0.573 (0.257, 0.783)	0.002	Moderate
Cross-Domain Correlations			
Pulsatility Index - TA Max Vel	-0.555 (-0.783, -0.190)	0.003	Moderate
Pulsatility Index - TA Mean Vel	-0.517 (-0.761, -0.144)	0.007	Moderate
ECM - VTI	-0.480 (-0.739, -0.097)	0.013	Moderate
TEX101 - VTI	-0.568 (-0.790, -0.209)	0.002	Moderate
